# Azithromycin in the Treatment of Preterm Prelabor Rupture of Membranes Demonstrates a Lower Risk of Chorioamnionitis and Postpartum Endometritis with an Equivalent Latency Period Compared with Erythromycin Antibiotic Regimens

**DOI:** 10.1155/2020/2093530

**Published:** 2020-07-09

**Authors:** Daniel Martingano, Shailini Singh, Antonina Mitrofanova

**Affiliations:** ^1^Department of Biomedical and Health Informatics, Rutgers University School of Health Professions, Newark, NJ, USA; ^2^Department of Obstetrics & Gynecology, St. John's Episcopal Hospital, Far Rockaway, NY, USA; ^3^Division of Maternal-Fetal Medicine, Newark Beth Israel Medical Center, Newark, NJ, USA; ^4^Department of Obstetrics & Gynecology, Rutgers Robert Wood Johnson Medical School, New Brunswick, NJ, USA

## Abstract

**Objective:**

To determine if antibiotic regimens including azithromycin versus erythromycin has an impact on pregnancy latency and development of clinical chorioamnionitis in the context of preterm prelabor rupture of membranes. *Study Design*. We conducted a prospective observational cohort study and followed all women receiving antibiotic regimens including either azithromycin or erythromycin in the context of preterm prelabor rupture of membranes. Primary outcomes were the duration of pregnancy latency period and development of chorioamnionitis. Secondary outcomes included neonatal sepsis with positive blood culture, cesarean delivery, postpartum endometritis, and meconium-stained amniotic fluid.

**Results:**

This study included 310 patients, with 142 receiving the azithromycin regimen and 168 receiving the erythromycin regimen. Patients receiving the azithromycin regimen had a statistically significant advantage in overall rates of clinical chorioamnionitis (13.4% versus 25%, *p* = 0.010), neonatal sepsis (4.9% versus 14.9%, *p* = 0.004), and postpartum endometritis (14.8% versus 31%, *p* = 0.001). In crude and adjusted models, when comparing the azithromycin group with the erythromycin group, a decreased risk was noted for the development of clinical chorioamnionitis, neonatal sepsis, and postpartum endometritis. Pregnancy latency by regimen was not significantly different in crude and adjusted models.

**Conclusion:**

Our study suggests that latency antibiotic regimens substituting azithromycin for erythromycin have lower rates and decreased risk of clinical chorioamnionitis, neonatal sepsis, and postpartum endometritis with no difference in pregnancy latency.

## 1. Introduction

Preterm prelabor rupture of membranes (PPROM) refers to prelabor rupture of membranes before 37 0/7 weeks of gestation. It is responsible for approximately one-third of preterm births and the single most common identifiable factor associated with preterm delivery [1–3]. The management of PPROM remains among the most controversial issues in maternal-fetal medicine. Remaining points of contention include duration and regimen of administration of antibiotic prophylaxis with goals of prolonging pregnancy latency and providing prophylaxis against maternal and fetal infection. The rationale for antibiotic prophylaxis is that infection appears to be both a cause and consequence of PPROM. Infection may lead to spontaneous preterm labor or may be the indication for medically indicated preterm delivery. The importance of reducing infection is highlighted by studies suggesting a relationship between chorioamnionitis, duration of membrane rupture, and development of cerebral palsy [4]. Currently, a regimen with reasonable activity against the major pelvic pathogens is employed for prophylaxis, but the optimal regimen is unclear.

The traditional and originally described regimen for prophylaxis of chorioamnionitis and increased pregnancy latency was outlined first by Mercer et al. [5] and involves intravenous ampicillin 2 g every 6 hours and erythromycin 250 mg every 6 hours for 48 hours followed by oral amoxicillin 250 mg every 8 hours and erythromycin 333 mg every 8 hours for five days. Presently, there are 3 retrospective studies [6–8] investigating the substitution of azithromycin for erythromycin in the setting of PPROM, each with unique conclusions and dosing strategies of each antibiotic: Pierson et al. [6] found no differences in latency from membrane rupture as well as no difference in secondary maternal and neonatal outcomes. Finneran et al. [7] also found no difference in pregnancy latency but noted a higher rate of cesarean delivery and positive neonatal blood cultures in those patients who received erythromycin for prophylaxis. Navathe et al. [8], when looking at patients who received a similar regimen to Pierson et al., Finneran et al., and this present study, found no differences in pregnancy latency, incidence of chorioamnionitis, or neonatal outcomes.

There are presently no prospective studies investigating the substitution of azithromycin for erythromycin in the setting of PPROM. The objective of this study was to determine if substituting azithromycin for erythromycin has an impact on pregnancy latency and development of chorioamnionitis in the context of PPROM.

## 2. Materials and Methods

We conducted a multicenter, prospective observational cohort study from July 2016 to November 2019 and followed all women receiving latency antibiotic treatment involving either azithromycin or erythromycin in the context of PPROM. Choice of regimen was determined by attending physician preference as either antibiotic was available at all sites involved. The azithromycin group consisted of azithromycin 1 g PO once and ampicillin 2 g every 6 hours IV for 48 hours followed by 5 days of amoxicillin 250 mg every 8 hours PO for 5 days. The erythromycin group consisted of erythromycin 250 mg and ampicillin 2 g every 6 hours IV for 48 hours followed by amoxicillin 250 mg and erythromycin 500 mg every 8 hours PO for 5 days.

Primary outcomes were the duration of pregnancy latency period and development of chorioamnionitis via either clinical or histologic criteria. Clinical chorioamnionitis was defined as outlined Higgins et al. [9]: by maternal temperature of 38° C or greater, without another source of fever, and with fetal tachycardia (greater than 160 beats per minute). Histological chorioamnionitis was confirmed on placental pathology examination postpartum.

Secondary outcomes included cesarean delivery, meconium-stained amniotic fluid, postpartum endometritis, and neonatal sepsis. Postpartum endometritis was defined as presence of a temperature of 38°C or greater, without another source of fever, and presence of fundal tenderness on physical examination in the postpartum period. Neonatal sepsis was defined as having a positive blood culture.

Patients were excluded if latency antibiotic regimen included combinations apart from those defined, if patients were less than 24 weeks gestational age, had been prescribed other antibiotics before presentation, had a cervical cerclage in situ, had a congenital or lethal fetal anomaly, or had a history of trauma or injury resulting in PPROM. All appropriate and required institutional approvals were obtained for this study.

### 2.1. Statistical Analysis

To account for unequal variance between treatment groups, Welch two-sample two-tailed *t*-test and Wilcoxon rank-sum test were used to evaluate differences between continuous variables as appropriate [10]. To evaluate differences between frequencies of binary variables between the treatment groups, we employed *X*^2^ test for comparing contingency tables as appropriate [11]. To examine treatment regimen effect on pregnancy latency, we employed Kaplan-Meier survival analysis [12]. To assess the contribution of each individual covariate on how it may effect the predictive ability of our model, we conducted the univariable Mantel-Cox proportional hazard model [13]. To further estimate the combined effect of all covariates, we performed multivariable Mantel-Cox proportional hazard model analysis. Relative risks of binary pregnancy outcomes (postpartum endometritis, cesarean delivery, chorioamnionitis, and meconium-stained amniotic fluid) between treatment groups (unadjusted and multivariable adjusted) were calculated using “modified Poisson regression”. This analysis was also conducted to determine risks of composite intra-amniotic infection/inflammation (III) and composite clinical infectious morbidity (CIM). III was defined as the presence of both clinical and/or histologic chorioamnionitis, and CIM was defined as the presence of both clinical chorioamnionitis and/or postpartum endometritis.

According to Zhou et al. [14], “modified Poisson regression” is defined as Poisson regression using robust error variance called sandwich estimation. Adjusted models were controlled for perceived confounding factors including maternal age, advanced maternal age, gestational age at diagnosis of PPROM, nulliparity, BMI, pregestational diabetes, group *β*-streptococcus positive (bacteriuria), any hypertensive disorder in pregnancy, and race.

We estimated the sample size a priori. Based upon previous studies [6–8], we estimated that 142 patients in each arm would be required for 99% power for continuous outcomes and 88% for binary outcomes to detect a 50% difference (*α* of 0.05 for a two-tailed test). Regarding the primary outcomes, this identified that our population would be powered to detect a noninferiority limit of 3 days of latency and 50% difference in chorioamnionitis. All statistics were performed using R version 3.4.0 [15].

## 3. Results

**Figure 1:**
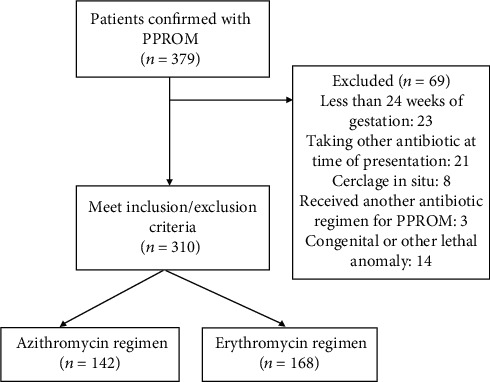
Flow chart of patient selection. 379 patients were confirmed with PPROM. 69 patients were excluded: 23 for being less than 24 weeks of gestation, 21 for taking other antibiotics at time of presentation, 8 with cerclages in situ, 3 who received another antibiotic regimen for PPROM, and 14 with a congenital or lethal anomaly. Of the 310 patients who met the inclusion/exclusion criteria, 142 received azithromycin while 168 received erythromycin.

This study included 310 patients, with 142 receiving the azithromycin regimen and 168 receiving the erythromycin regimen (Figure 1). Maternal demographics included maternal age, advanced maternal age (AMA), gestational age at initial diagnosis of PPROM, BMI, pregestational diabetes, group *β*-streptococcus positive (bacteriuria), any hypertensive disorder in pregnancy (including chronic hypertension, gestational hypertension, or preeclampsia with or without severe features), and race. Maternal demographics are shown in Table 1. A statistically significant difference was noted in percentage of nulliparous patients with *X*^2^*p* value of 0.042 with all other demographic components being not significantly different.

Comparison of pregnancy and neonatal outcomes between treatment groups is shown in Table 2. Regarding the primary outcomes, we observed there was no difference in pregnancy latency and significant differences in the rates of clinical chorioamnionitis, but not histologic chorioamnionitis. The difference in the latency period between treatment groups was median of 5 days, interquartile range (IQR) (6–11) days for the azithromycin group versus 4.5 days, IQR (6–10.8) for erythromycin group (*p* = 0.836, Wilcoxon Rank-sum Test). Rates of clinical chorioamnionitis were significantly lower in the azithromycin group versus the erythromycin group (13.4% versus 25%, *p* = 0.035, *X*^2^ test) while rates of histologic chorioamnionitis were not significantly different between groups (69.7% in the azithromycin group versus 64.9% in the erythromycin group, *p* = 0.367, *X*^2^ test).

Regarding secondary outcomes, statistically significant differences were noted in the rates of postpartum endometritis, neonatal sepsis, and meconium-stained amniotic fluid. No differences were noted in rates of cesarean delivery. The azithromycin group was noted to have lower rates of postpartum of endometritis at 14.8% versus 31% in the erythromycin group, *p* = <0.001, *X*^2^ test. Rates of meconium-stained amniotic fluid were noted to be higher in the azithromycin group as compared with the erythromycin group at a rate of 26.8% versus 15.15%, *p* = 0.14. Rates of neonatal sepsis were higher in the azithromycin group as compared with the azithromycin group at a rate of 4.9% versus 14.9%, *p* = 0.004, *X*^2^ test).

**Figure 2:**
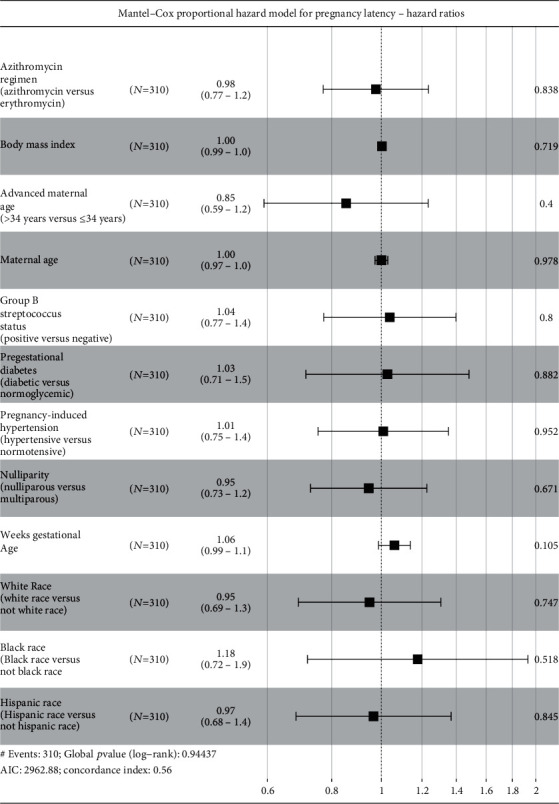
Mantel-Cox proportional hazard model with hazard ratios. This analysis demonstrated that all covariates in isolation did not effect pregnancy latency in the multivariable (adjusted) model.

**Figure 3:**
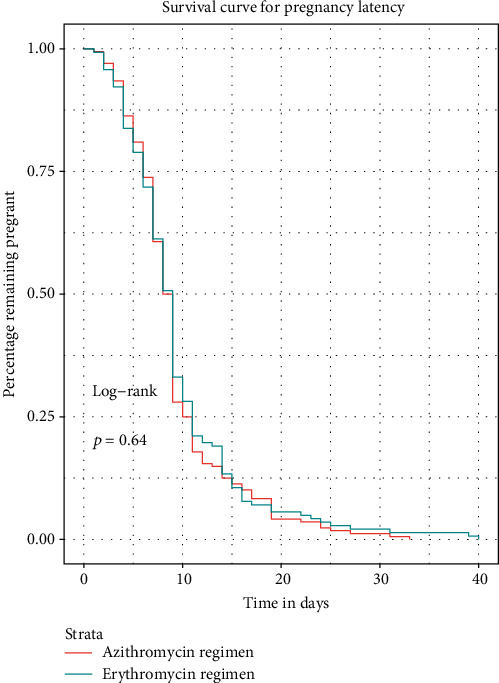
Survival curve for pregnancy latency. This demonstrated no difference in pregnancy latency between treatment types (*p* = 0.64, log-rank test).

**Figure 4:**
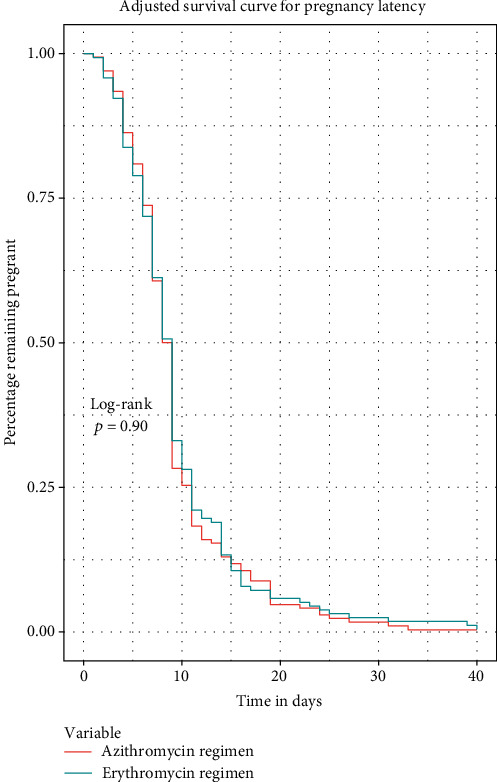
Adjusted survival curve for pregnancy latency. After controlling for covariates, the adjusted survival curves by treatment type were not noted to be significantly different (*p* = 0.90, log-rank test).

Effect of treatment regimens on pregnancy latency using multivariable (adjusted) analysis with the Mantel-Cox proportional hazard model with hazard ratios are shown graphically in Figure 2. This analysis demonstrated that all covariates in isolation did not effect pregnancy latency in the multivariable (adjusted) model. Unadjusted and adjusted Kaplan-Meier survival analyses regarding pregnancy latency are shown in Figures 3 and 4, respectively. This demonstrated no difference in pregnancy latency between treatment types (*p* = 0.64, log-rank test). After controlling for covariates, the adjusted survival curves by treatment type were not noted to be significantly different (*p* = 0.90, log-rank test).

Crude and adjusted risk ratios for binary pregnancy outcomes including chorioamnionitis, cesarean delivery, meconium-stained amniotic fluid, neonatal sepsis, and postpartum endometritis are shown in Table 3. In crude analysis, a 46% lower risk of clinical chorioamnionitis was noted in the azithromycin group with a 49% decreased risk after adjusted analysis, *p* = 0.024 and 0.015, respectively. Regarding postpartum endometritis, a 52% decreased risk was noted for the azithromycin group in crude analysis and a 54% decreased risk in the adjusted analysis, *p* = 0.004 and 0.002, respectively. Regarding neonatal sepsis, a 67% decreased risk was noted for the azithromycin group in crude analysis and a 68% decreased risk in the adjusted analysis, *p* = 0.010 and 0.005, respectively. Regarding meconium-stained amniotic fluid, a 73% increased risk was noted for the azithromycin group in crude analysis and a 69% increased risk in the adjusted analysis, *p* = 0.031 and 0.042, respectively. Regarding CIM, a 50% decreased risk was noted in the azithromycin group in crude analysis and a 52% decreased risk in adjusted analysis, *p* = <0.001 for both. All other outcomes including histologic chorioamnionitis, III, and cesarean delivery did not achieve a statistically significant difference in either crude or adjusted models.

Given statistical significance of percentage of nulliparous patients between treatment groups, crude and adjusted risk ratios were conducted for this population of patients within treatment groups in the same manner as noted above and are shown in Table 4. Of the 218 nulliparous patients present in this study, 108 patients received azithromycin while 110 received erythromycin. When comparing these two groups, the significant decreased risks in clinical chorioamnionitis, postpartum endometritis, CIM, and neonatal sepsis were similarly noted as in the entire study population with the exception of meconium-stained amniotic fluid. In crude analysis, a 57% lower risk of clinical chorioamnionitis was noted in the azithromycin group, with a 61% decreased risk after adjusted analysis, *p* = 0.006 and 0.003, respectively. Regarding postpartum endometritis, a 50% decreased risk was noted for the azithromycin group in crude analysis and a 53% decreased risk in the adjusted analysis, *p* = 0.012 and 0.010, respectively. Regarding neonatal sepsis, an 81% decreased risk was noted for the azithromycin group in crude analysis and a 93% decreased risk in the adjusted analysis, *p* ≤ 0.001 for both, respectively. Regarding CIM, a 53% decreased risk was noted in the azithromycin group in crude analysis and a 57% decreased risk in adjusted analysis, *p* ≤ 0.001 for both. All other outcomes including histologic chorioamnionitis, III, and cesarean delivery did not achieve a statistically significant difference in either crude or adjusted models, similar to the results of the entire study population. Therefore, the significant difference in the percentage of nulliparous patients between treatment groups does not affect the overall conclusions in treatment outcomes.

Detailed multivariable analyses for outcomes achieving a statistical significance via modified Poisson linear regression are shown in Supplemental Tables see (available here); all of which did not achieve statistically significant differences. Univariable analysis of the pregnancy outcomes in relation to the factor of nulliparity, given its statistical significance between treatment groups, is shown in Supplemental Tables (available here) and was noted to have a significant effect in composite intra-amniotic infection/inflammation and histologic chorioamnionitis.

## 4. Discussion

This is the first prospective study to evaluate difference in antibiotic regimens substituting azithromycin for erythromycin. Our study suggests that latency antibiotic regimens substituting azithromycin for erythromycin have a significantly lower rates and risk of clinical chorioamnionitis, neonatal sepsis, postpartum endometritis, and CIM, with an increased rate and risk of meconium-stained amniotic fluid.

Regarding our primary outcomes, our finding of no differences in pregnancy latency is consistent with the previous studies by Pierson et al., Finneran et al., and Navathe et al. Our results differ from the previous three studies in regards to rates and risk of clinical chorioamnionitis where we found significantly lower rates for the azithromycin versus erythromycin regimens. However, when evaluating for III, our study found no differences between groups, which is consistent with prior studies.

Regarding secondary outcomes, our findings of lower rates of neonatal sepsis is consistent with Finneran et al. but differs from Pierson et al. and Navathe et al. Our results demonstrate rates similar to Finneran et al. in terms of neonatal sepsis via positive blood culture and lower rates when substituting azithromycin for erythromycin. This congruence strengthens the possibility that substitution of azithromycin for erythromycin may provide neonatal benefit. Our findings, both before and after adjusting for confounding factors, of a lower rate and risk of clinical chorioamnionitis are consistent with our additional finding of lower rates and risk of postpartum endometritis, likely given the association of both clinical scenarios and indeed is reflected in the significant difference of CIM between the two treatment groups. The finding of increased rates and risk of meconium-stained amniotic fluid is of undetermined significance.

We believe these findings to be generalizable across different races and varied geographical settings given that both patient populations were similarly racially diverse. Our populations, however, were predominantly White (43% and 42%) and Hispanic race (36% and 42%), and thus, the results of our study are most applicable to patients of those races. This difference in race may additionally contribute to the differences in results from previous studies where our study had a larger representation of Hispanic race as compared to Pierson et al. who had predominantly White (48% and 43%) and Black (32% and 32.3%) race, Finneran et al. who had predominantly Black (47.6% and 44.9%) and White (28.6% and 26.9%), and Navathe et al. who (in the single-dose azithromycin arm) had predominantly Black race (43.5% and 41.0%).

This study was additionally limited in that the antibiotic regimens were decided solely on physician preference. The preference for latency antibiotic regimen was determined by the covering attending physician at the time of diagnosis. No specific analysis was performed regarding specific physician preference and remains a limitation of this study.

Other regimens employed for the management of PPROM were not considered, which prohibits a more robust analysis of which antibiotic regimen is indeed the most beneficial. Our institution does not use regimens apart from those included in the study and thus is limited on that basis. Through our robust Kaplan-Meier and Mantel-Cox analysis comparing difference in treatment types and specific covariates within those treatment types, we can conclude with confidence that in this prospective study, the type of antibiotic regimen did not make a difference in pregnancy latency and that this effect was not confounded by other factors, which is in agreement with the conclusions of previous studies that stated there was no difference noted in pregnancy latency.

## 5. Conclusion

Antibiotic regimens substituting azithromycin for erythromycin appear to have no difference in pregnancy latency yet demonstrates significantly lower rates and decreased risk of neonatal sepsis, clinical chorioamnionitis, and postpartum endometritis and an increased risk of meconium-stained amniotic fluid. Further studies are required to elucidate the differences in the findings of this prospective study to that of previous retrospective studies. Given that all studies performed do not demonstrate an inferiority of azithromycin substitution over erythromycin, utilizing this substitution remains a reasonable option in the management of PPROM.

## Figures and Tables

**Table 1:** Maternal demographics.

Characteristic	Azithromycin group *n* = 142	Erythromycin group *n* = 168	*p*
Maternal age (years)	32 ± 5.3 (20-43)	31.5 ± 5.8 (18-47)	0.410^∗^
Advanced maternal age	49 (35)	45 (27)	0.104^*δ*^
Gestational age at diagnosis	29.9 ± 1.76 (25-33)	30.2 ± 1.71 (26-33)	0.380^∗^
Nulliparous	108 (76.1)	110 (65.5)	0.042^*δ*^
BMI (kg/m^2^)	32.8 ± 5.1 (21-48.9)	33.86 ± 4.1 (20.8-43.9)	0.282^∗^
Pregestational diabetes	15 (10.6)	20 (11.9)	0.153^*δ*^
Group *β*-streptococcus positive	28 (19.7)	38 (22.6)	0.534^*δ*^
Hypertensive disorders in pregnancy	29 (20.4)	41 (24.4)	0.403^*δ*^
Race			
White	60 (42.3)	74 (44)	0.832^*δ*^
Asian	30 (21.1)	31 (18.5)	0.555^*δ*^
Hispanic	37 (26.7)	51 (30.4)	0.486^*δ*^
Black	14 (9.9)	13 (7.1)	0.509^*δ*^

Data are presented as mean ± standard deviation (range) or n (%). ^*δ*^Statistics performed using *X*^2^ test. ^∗^Statistics performed using Welch two-sample *t*-test.

**Table 2:** Pregnancy and neonatal outcomes.

Characteristic	Azithromycin group *n* = 142	Erythromycin group *n* = 168	*p*
Clinical chorioamnionitis	19 (13.4)	42 (25)	0.010
Histological chorioamnionitis	99 (69.7)	109 (64.9)	0.367
Latency interval	5 (6-11)	4.75 (6 – 10.8)	0.836
Cesarean delivery	76 (50.7)	83 (49.4)	0.470
Meconium-stained amniotic fluid	38 (26.8)	26 (15.5)	0.014
Postpartum endometritis	21 (14.8)	52 (31)	<0.001
Neonatal sepsis	7 (4.9)	25 (14.9)	0.004

Data are presented as median (interquartile range) or *n* (%). ^*δ*^Statistics performed using *X*^2^ test. ^∗^Statistics performed using the Wilcoxon rank-sum test.

**Table 3:** Crude and adjusted risk ratios for pregnancy outcomes for azithromycin versus erythromycin regimens.

	Crude			Adjusted∗		
	RR	95% CI	*p* ^+^	RR	95% CI	*p* ^+^
Clinical chorioamnionitis	0.54	0.31 to 0.92	0.024	0.51	0.30 to 0.89	0.015
Histologic chorioamnionitis	1.07	0.82 to 1.41	0.604	1.08	0.82 to 1.43	0.569
Postpartum endometritis	0.48	0.29 to 0.79	0.004	0.46	0.27 to 0.76	0.002
Composite intra-amniotic infection/inflammation	0.92	0.73 to 1.18	0.523	0.92	0.72 to 1.18	0.524
Composite clinical infectious morbidity	0.50	0.35 to 0.73	<0.001	0.48	0.33 to 0.70	<0.001
Neonatal sepsis (positive blood culture)	0.33	0.14 to 0.77	0.010	0.32	0.14 to 0.76	0.005
Cesarean delivery	1.08	0.79 to 1.48	0.614	1.05	0.76 to 1.44	0.774
Meconium-stained amniotic fluid	1.73	1.05 to 2.85	0.031	1.69	1.01 to 2.81	0.042

^∗^Models were adjusted for maternal age, advanced maternal age, gestational age at diagnosis of PPROM, nulliparity, BMI, pregestational diabetes, group *ß*-streptococcus positive (bacteriuria), any hypertensive disorder in pregnancy, and race. ^+^*p* values calculated using the likelihood ratio test. Estimates are calculated via modified Poisson generalized linear models. RR = risk ratio; CI = confidence interval.

**Table 4:** Crude and adjusted risk ratios for pregnancy outcomes for azithromycin versus erythromycin regimens for nulliparous patients.

	Crude			Adjusted∗		
	RR	95% CI	*p* ^+^	RR	95% CI	*p* ^+^
Clinical chorioamnionitis	0.43	0.23 to 0.81	0.006	0.39	0.20 to 0.75	0.003
Histologic chorioamnionitis	0.96	0.70 to 1.30	0.784	0.94	0.69 to 1.30	0.724
Postpartum endometritis	0.50	0.28 to 0.87	0.012	0.47	0.26 to 0.85	0.010
Composite intra-amniotic infection/inflammation	0.81	0.62 to 1.06	0.127	0.78	0.59 to 1.04	0.092
Composite clinical infectious morbidity	0.47	0.31 to 0.71	<0.001	0.43	0.28 to 0.67	<0.001
Neonatal sepsis (positive blood culture)	0.19	0.07 to 0.57	<0.001	0.20	0.07 to 0.59	<0.001
Cesarean delivery	1.19	0.81 to 1.76	0.376	1.24	0.83 to 1.85	0.299
Meconium-stained amniotic fluid	1.48	0.84 to 2.61	0.176	1.40	0.78 to 2.52	0.263

^∗^Models were adjusted for maternal age, advanced maternal age, gestational age at diagnosis of PPROM, nulliparity, BMI, pregestational diabetes, group *ß*-streptococcus positive (bacteriuria), any hypertensive disorder in pregnancy, and race. ^+^*p* values calculated using the likelihood ratio test. Estimates are calculated via modified Poisson generalized linear models. RR = risk ratio; CI = confidence interval.

## Data Availability

Presently, this data is not available for sharing at this time due to institutional safety and privacy regulations.

## Supplementary Materials

Supplementary Materials Supplemental Table 1: multivariable analysis for clinical chorioamnionitis. Supplemental Table 2: multivariable analysis for postpartum endometritis. Supplemental Table 3: multivariable analysis for composite clinical infectious morbidity. Supplemental Table 4: multivariable analysis for neonatal sepsis. Supplemental Table 5: univariable analysis for nulliparity on pregnancy outcomes.
