# Supplementary material for: Azithromycin in the Treatment of Preterm Prelabor Rupture of Membranes Demonstrates a Lower Risk of Chorioamnionitis and Postpartum Endometritis with an Equivalent Latency Period Compared with Erythromycin Antibiotic Regimens
Source: Infect Dis Obstet Gynecol. 2020 Jul 9;2020:2093530. doi: 10.1155/2020/2093530 (PMC7368187; doi:10.1155/2020/2093530)
Supplement: Supplementary Materials — Supplemental Table 1: multivariable analysis for clinical chorioamnionitis. Supplemental Table 2: multivariable analysis for postpartum endometritis. Supplemental Table 3: multivariable analysis for composite clinical infectious morbidity. Supplemental Table 4: multivariable analysis for neonatal sepsis. Supplemental Table 5: univariable analysis for nulliparity on pregnancy outcomes. [file 2093530.f1.docx]

| Supplemental Table 1. Multivariable Analysis for Clinical Chorioamnionitis | | | | | | |  |
| --- | --- | --- | --- | --- | --- | --- | --- |
|  | **Crude** | | |  | **Adjusted*** | | |
|  | **RR** | **95% CI** | ***p^+^*** |  | **RR** | **95% CI** | ***p^+^*** |
| Regimen | 0.54 | 0.31 to 0.92 | 0.024 |  | 0.51 | 0.30 to 0.89 | 0.015 |
| Body-Mass Index | 1.02 | 0.99 to 1.05 | 0.280 |  | 1.02 | 0.99 to 1.05 | 0.276 |
| Advanced Maternal Age | 0.82 | 0.47 to 1.44 | 0.496 |  | 0.90 | 0.41 to 1.98 | 0.789 |
| Maternal Age | 0.99 | 0.949 to 1.04 | 0.729 |  | 1.00 | 0.94 to 1.07 | 0.935 |
| Gestational Age at Diagnosis of PPROM | 1.08 | 0.93 to 1.26 | 0.311 |  | 1.10 | 0.95 to 1.28 | 0.187 |
| Group ß-streptococcus positive (bacteriuria) | 0.48 | 0.22 to 1.05 | 0.067 |  | 0.48 | 0.21 to 1.07 | 0.051 |
| Pregestational Diabetes | 0.55 | 0.20 to 1.52 | 0.250 |  | 0.71 | 0.25 to 1.99 | 0.494 |
| Hypertensive Disorders in Pregnancy | 0.75 | 0.39 to 1.45 | 0.397 |  | 0.64 | 0.33 to 1.25 | 0.175 |
| Nulliparity | 1.42 | 0.78 to 2.57 | 0.253 |  | 1.57 | 0.85 to 2.89 | 0.134 |
| Race | 0.98 | 0.75 to 1.26 | 0.850 |  | 1.06 | 0.81 to 1.38 | 0.682 |
| *maternal age, advanced maternal age, gestational age at diagnosis of PPROM, nulliparity, BMI, pregestational diabetes, group ß-streptococcus positive (bacteriuria), any hypertensive disorder in pregnancy, and race  ^+^*p* values calculated using Likelihood Ratio Test  Estimates are calculated via modified Poisson generalized linear models.  RR=risk ratio, CI=confidence interval. | | | | | | |  |

| Supplemental Table 2. Multivariable Analysis for Postpartum Endometritis | | | | | | |  |
| --- | --- | --- | --- | --- | --- | --- | --- |
|  | **Crude** | | |  | **Adjusted*** | | |
|  | **RR** | **95% CI** | ***p^+^*** |  | **RR** | **95% CI** | ***p^+^*** |
| Regimen | 0.48 | 0.29 to 0.79 | 0.004 |  | 0.46 | 0.27 to 0.76 | 0.002 |
| Body-Mass Index | 0.99 | 0.97 to 1.02 | 0.550 |  | 0.99 | 0.96 to 1.02 | 0.495 |
| Advanced Maternal Age | 0.80 | 0.48 to 1.35 | 0.406 |  | 1.11 | 0.54 to 2.30 | 0.774 |
| Maternal Age | 0.98 | 0.94 to 1.02 | 0.248 |  | 0.97 | 0.92 to 1.03 | 0.359 |
| Gestational Age at Diagnosis of PPROM | 1.07 | 0.93 to 1.23 | 0.321 |  | 1.09 | 0.94 to 1.25 | 0.241 |
| Group ß-streptococcus positive (bacteriuria) | 0.73 | 0.39 to 1.35 | 0.313 |  | 0.69 | 0.37 to 1.30 | 0.237 |
| Pregestational Diabetes | 0.58 | 0.23 to 1.43 | 0.236 |  | 0.72 | 0.29 to 1.79 | 0.454 |
| Hypertensive Disorders in Pregnancy | 0.96 | 0.55 to 1.68 | 0.892 |  | 0.84 | 0.47 to 1.48 | 0.533 |
| Nulliparity | 1.29 | 0.76 to 2.2 | 0.349 |  | 1.41 | 0.82 to 2.43 | 0.203 |
| Race | 1.1 | 0.88 to 1.38 | 0.39 |  | 1.15 | 0.91 to 1.45 | 0.253 |
| *maternal age, advanced maternal age, gestational age at diagnosis of PPROM, nulliparity, BMI, pregestational diabetes, group ß-streptococcus positive (bacteriuria), any hypertensive disorder in pregnancy, and race  ^+^*p* values calculated using Likelihood Ratio Test  Estimates are calculated via modified Poisson generalized linear models.  RR=risk ratio, CI=confidence interval. | | | | | | |  |

| Supplemental Table 3. Multivariable Analysis for Composite Clinical Infectious Morbidity | | | | | | |  |
| --- | --- | --- | --- | --- | --- | --- | --- |
|  | **Crude** | | |  | **Adjusted*** | | |
|  | **RR** | **95% CI** | ***p^+^*** |  | **RR** | **95% CI** | ***p^+^*** |
| Regimen | 0.50 | 0.35 to 0.73 | <0.001 |  | 0.48 | 0.33 to 0.70 | <0.001 |
| Body-Mass Index | 1.00 | 0.983 to 1.02 | 0.773 |  | 1.00 | 0.98 to 1.02 | 0.821 |
| Advanced Maternal Age | 0.81 | 0.56 to 1.19 | 0.284 |  | 1.01 | 0.59 to 1.72 | 0.970 |
| Maternal Age | 0.98 | 0.95 to 1.01 | 0.277 |  | 0.99 | 0.94 to 1.03 | 0.527 |
| Gestational Age at Diagnosis of PPROM | 1.08 | 0.97 to 1.19 | 0.157 |  | 1.10 | 0.99 to 1.21 | 0.078 |
| Group ß-streptococcus positive (bacteriuria) | 0.61 | 0.38 to 0.99 | 0.047 |  | 0.60 | 0.36 to 0.98 | 0.031 |
| Pregestational Diabetes | 0.57 | 0.29 to 1.11 | 0.099 |  | 0.71 | 0.36 to 1.41 | 0.308 |
| Hypertensive Disorders in Pregnancy | 0.87 | 0.57 to 1.32 | 0.501 |  | 0.74 | 0.48 to 1.15 | 0.170 |
| Nulliparity | 1.35 | 0.9 to 2.0 | 0.143 |  | 1.48 | 0.99 to 2.22 | 0.052 |
| Race | 1.04 | 0.88 to 1.24 | 0.612 |  | 1.11 | 0.93 to 1.32 | 0.262 |
| *maternal age, advanced maternal age, gestational age at diagnosis of PPROM, nulliparity, BMI, pregestational diabetes, group ß-streptococcus positive (bacteriuria), any hypertensive disorder in pregnancy, and race  ^+^*p* values calculated using Likelihood Ratio Test  Estimates are calculated via modified Poisson generalized linear models.  RR=risk ratio, CI=confidence interval. | | | | | | |  |

| Supplemental Table 4. Multivariable Analysis for Neonatal Sepsis | | | | | | |  |
| --- | --- | --- | --- | --- | --- | --- | --- |
|  | **Crude** | | |  | **Adjusted*** | | |
|  | **RR** | **95% CI** | ***p^+^*** |  | **RR** | **95% CI** | ***p^+^*** |
| Regimen | 0.33 | 0.14 to 0.77 | 0.010 |  | 0.32 | 0.14 to 0.76 | 0.005 |
| Body-Mass Index | 1.01 | 0.97 to 1.06 | 0.535 |  | 1.01 | 0.97 to 1.05 | 0.637 |
| Advanced Maternal Age | 0.71 | 0.32 to 1.58 | 0.402 |  | 1.06 | 0.35 to 3.16 | 0.919 |
| Maternal Age | 0.97 | 0.92 to 1.04 | 0.398 |  | 0.97 | 0.89 to 1.06 | 0.522 |
| Gestational Age at Diagnosis of PPROM | 1.03 | 0.84 to 1.27 | 0.742 |  | 1.05 | 0.89 to 1.29 | 0.624 |
| Group ß-streptococcus positive (bacteriuria) | 0.38 | 0.12 to 1.26 | 0.113 |  | 0.40 | 0.12 to 1.36 | 0.103 |
| Pregestational Diabetes | 0.52 | 0.13 to 2.19 | 0.376 |  | 0.80 | 0.19 to 3.45 | 0.762 |
| Hypertensive Disorders in Pregnancy | 0.96 | 0.42 to 2.22 | 0.924 |  | 0.92 | 0.39 to 2.18 | 0.848 |
| Nulliparity | 1.51 | 0.65 to 3.48 | 0.337 |  | 1.64 | 0.69 to 3.88 | 0.241 |
| Race | 0.69 | 0.45 to 1.04 | 0.073 |  | 0.72 | 0.46 to 1.1 | 0.109 |
| *maternal age, advanced maternal age, gestational age at diagnosis of PPROM, nulliparity, BMI, pregestational diabetes, group ß-streptococcus positive (bacteriuria), any hypertensive disorder in pregnancy, and race  ^+^*p* values calculated using Likelihood Ratio Test  Estimates are calculated via modified Poisson generalized linear models.  RR=risk ratio, CI=confidence interval. | | | | | | |  |

| Supplemental Table 5. Univariable Analysis for Nulliparity on Pregnancy Outcomes | | | | | |
| --- | --- | --- | --- | --- | --- |
|  |  | | |  |  |
|  | **RR** | **95% CI** | ***p^+^*** |  |  |
| Clinical Chorioamnionitis | 1.42 | 0.78 to 2.57 | 0.253 |  |  |
| Histologic Chorioamnionitis | 1.53 | 1.10 to 2.13 | 0.009 |  |  |
| Postpartum Endometritis | 1.29 | 0.76 to 2.20 | 0.339 |  |  |
| Composite Intraamniotic Infection/Inflammation | 1.50 | 1.13 to 2.01 | 0.004 |  |  |
| Composite Clinical Infectious Morbidity | 1.35 | 0.90 to 2.00 | 0.134 |  |  |
| Neonatal Sepsis (Positive Blood Culture) | 1.51 | 0.65 to 3.48 | 0.320 |  |  |
| Cesarean Delivery | 0.76 | 0.55 to 1.04 | 0.094 |  |  |
| Meconium-Stained  Amniotic Fluid | 1.38 | 0.77 to 2.46 | 0.264 |  |  |
| ^+^*p* values calculated using Likelihood Ratio Test  Estimates are calculated via modified Poisson generalized linear models.  RR=risk ratio, CI=confidence interval. | | | | | |
